# High functional conservation of *takeout* family members in a courtship model system

**DOI:** 10.1371/journal.pone.0204615

**Published:** 2018-09-27

**Authors:** Sumit Saurabh, Nancy Vanaphan, Walter Wen, Brigitte Dauwalder

**Affiliations:** Department of Biology and Biochemistry, University of Houston, Houston, Texas, United States of America; New Mexico State University, UNITED STATES

## Abstract

*takeout* (*to*) is one of the male-specific genes expressed in the fat body that regulate male courtship behavior, and has been shown to act as a secreted protein in conjunction with courtship circuits. There are 23 *takeout* family members in *Drosophila melanogaster*, and homologues of this family are distributed across insect species. Sequence conservation among family members is low. Here we test the functional conservation of *takeout* family members by examining whether they can rescue the *takeout* courtship defect. We find that despite their sequence divergence *takeout* members from *Aedes aegypti* and *Epiphas postvittana*, as well as family members from *D*. *melanogaster* can substitute for *takeout* in courtship, demonstrating their functional conservation. Making use of the known *E*. *postvittana* Takeout structure, we used homology modeling and amphipathic helix analysis and found high overall structural conservation, including high conservation of the structure and amphipathic lining of an internal cavity that has been shown to accommodate hydrophobic ligands. Together these data suggest a high degree of structural conservation that likely underlies functional conservation in courtship. In addition, we have identified a role for a conserved exposed protein motif important for the protein’s role in courtship.

## Introduction

Courtship rituals in *Drosophila melanogaster* consist of a series of stereotyped behaviors displayed by the male in order to gain access to and mate with females [1, 2]. This behavior is regulated by the general sex determination pathway that controls sex-specific expression of the two master regulators *doublesex* (*dsx*) and *fruitless* (*fru*) [3–6]. Little is known how their downstream target genes control mating behavior. One of them, *takeout (to)*, is regulated by both *dsx and fru* [7] and has been studied in some detail. Mutations in *takeout* result in reduced male courtship behavior. Mutant males are capable of all steps of courtship, but display them with reduced frequency [7]. *takeout* is male-specifically expressed in the head fat body, from where it is secreted into the hemolymph and acts as a secreted protein [8]. *takeout* has the characteristics of small soluble proteins and is most similar to Juvenile Hormone Binding Proteins (JHBPs) from other insects. In addition to expression in the fat body, *takeout* is also expressed in the antennae in both sexes [7]. *takeout* has been shown to have a function in the larval response to starvation, and *to* mutant larvae were observed to die early in response to food deprivation [7, 9]. Furthermore, *takeout* has also been implicated in the control of aging and longevity. The To protein was found to be up-regulated in flies with extended lifespans and data from dietary restrictions-based lifespan screens support a role for To in *D*. *melanogaster* aging physiology, as To concentrations were found to correlate with extended lifespan [10, 11].

Takeout is the founding member of a newly identified gene family. Twenty-three homologs of *takeout* have been identified in *D*. *melanogaster* [7, 9, 12]. With the exception of two conserved motifs the sequence conservation between family members is fairly low, the most distant paralog being only 18% identical (CG16820). Except for *takeout*, their specific functions are unknown, however several exhibit circadian regulated expression, and all contain signal sequences indicative of secreted proteins. In addition to *takeout*, we have shown that other homologs exhibit male-specific expression [7, 13]. *takeout* homologs have been identified in several other insect species in a variety of tissues, including olfactory organs [7–9, 12, 14–20]. Together, current data suggest that *takeout* is part of a large gene family found throughout insects with roles in metabolism, circadian behavior, aging, and male courtship behavior.

A comprehensive phylogenetic analysis of *takeout* gene family members across 21 species of insects grouped To family members in separate clusters /clades. Each To member can be assigned to a specific clade based on sequence similarity. This suggests that To might have evolutionary conserved roles. A comparison of To family members from different species suggests that this family of proteins is old and duplication of TO genes preceded speciation. But we also observed many instances of gene duplication and loss and evidence of positive selection in several lineages [13], consistent with the action of sexual selection on male-specifically expressed genes. These findings raise the possibility that the *takeout* gene family is a group of conserved proteins that may have maintained similar functional roles across species among at least some of its members. In this work, we test this hypothesis by focusing on the courtship phenotype of *D*. *mel*. *takeout* mutants and ask whether *takeout* homologues from other species and from *D*. *mel*. are capable of rescuing the courtship defect. We find that the tested members can substitute for *takeout* despite their relatively low sequence conservation. We use homology modeling to compare *D*. *mel*. Takeout protein structure with the structure of a previously crystallized Takeout protein from *Epiphyas postvittana* [19, 21] and find high structural conservation as a possible unifying functional feature among family members.

## Materials and methods

### Fly strains

Fly strains were reared on standard sugar-based corn meal medium at 25°C under a controlled 12hr-12hr light dark cycle. The fat body specific *Lsp2-Gal4* (on the 2^nd^ chromosome) used in this study was established by mobilizing *Lsp2-Gal4* from our previous *Lsp2-Gal4 strain* (with insert on the 3^rd^ Chromosome) [8]. *UAS-13618* and *UAS-16820* were established in the lab by PCR amplification from head cDNA. *UAS-A*. *aegypti To*, *UAS-D*.*mel To*, *UAS-Ep*.*To*, *UAS-B*.*mori JHBP* and *UAS-D*.*mel To-mut* were established during this study by PCR using the primers indicated. All constructs were constructed with a V5 protein tag at the C-terminus. All primers used are listed below. *Aedes aegypti* cDNA was prepared from head RNA kindly provided by Dr. David Severson, University of Notre Dame. *Epiphyas postvittana* To and *B*. *mori* JHBP were amplified from plasmids kindly provided by Dr. Cyril Hamiaux, The New Zealand Institute for Plant & Food Research Limited, and Dr. Toshimasa Yamazaki, National Institute of Agrobiological Sciences, Japan, respectively. Primers were designed with Not1(5’ *GCGGCCGC* 3’) and Xba1(5’ *TCTAGA* 3’) restriction sites at their 5’ and 3’ ends respectively (Table 1). Constructs were inserted as *Not1*/*Xba1* fragments into the *pUAST-attB* transformation vector. All constructs were sequenced and sent to Rainbow Genetics, Inc. for injection. All plasmids were inserted on the second chromosome at the attP-VK22 site (PBac{y^+^-attP-9A} VK00022).

**Table 1:** Primers used to generate constructs.

V5-Tag	5’ CGTAGAATCGAGACCGAGGAGAGGGTTAGGGATAGGCTTACC 3’
Aedes To-F	5’ GGCGGCCGCCATGAAGGTCATGGTTAGTGGAACAG3’
Aedes-ToV5 -R	5’ GTCTAGACTTACGTAGAATCGAGACCGAGGAGAGGGTTAGGGATAGGCTT ACCCTGGAACAGTTCGTTGTAAGGCACCTT 3’
CG16820-F	5’ GGCGGCCGCCATGCAATTGCCTTGCATATCGCTC 3’
CG16820V5-R	5’GTCTAGACTTACGTAGAATCGAGACCGAGGAGAGGGTTAGGGATAGGCTTACCTTGAACCAAGAATTTCTCGATGGGTACCTT 3’
E. postvittana-F	5’ GGCGGCCGCCATGACCTCGGCTTTCCAGCAGGCG 3’
E. postvittanaV5-R	5’ GTCTAGACTTACGTAGAATCGAGACCGAGGAGAGGGTTGGGATAGGCTT ACCTACATTAGCAATTTCTGCTATCGGCACTTT 3’
JHBP-F	5’ GGCGGCCGCCATGGCTTCTTTGAAAGTATTCCTGG 3’
JHBP-R	5’ GTCTAGACTTACGTAGAATCGAGACCGAGGAGAGGGTTAGGGATAGGCTT ACCATTAAGATTTTCGAAGAAGCTTGACGCGGG 3’
pUAST-F	5’ GCAACTACTGAAATCTGCCAAG 3’
pUAST-R	5’ TTGTCCAATTATGTCACACCACAGA 3’

### Multiple alignment and complementation analysis

Transgenes used for complementation analysis were analyzed and displayed as cladogram using NCBI-COBALT (Constraint Based Multiple Alignment Tool). Scale bar length represents number of amino acid substitutions per site.

Ramachandran Plot Analysis was performed on the modelled Takeout structure using MOLPROBITY [22]). Most residues were found in favorable positions. 95.5% (211 of 221) of all residues were in favored regions. Two outliers were present but not in the relevant motifs (105 ARG, 109 ALA). 99.1% of residues were in regions that were allowed.

### Modeling and site directed mutagenesis

Sequences were compared using the constraint based multiple alignment tool (NCBI) and PRofile ALIgNEment (PRALINE -http://www.ibi.vu.nl/programs/pralinewww/). The *Dmel*-To sequence was modelled onto the *E*.*postvittana*-To structure (Protein Databank ID– 3E8T) using SWISS-MODEL (BIOZENTRUM). The modelled structure with highest QMEAN4 score was chosen and conserved residues were mapped and displayed using UCSF-CHIMERA protein. The highly conserved motif2 was found to be at the exterior of the protein. Conserved residues from this motif were chosen for mutagenesis (**N**l**FN**gdkalg**D**nmnv**F**lnen). Mutagenesis was performed using Agilent’s Quick Change Site directed mutagenesis kit in two rounds following the supplier’s instructions. Primers used are indicated below (Table 2). The mutated sequence was inserted into *pUAST-attP*, sequenced and injected into the attP-*Drosophila* line VK22 as described above.

**Table 2:** Primers used for mutagenesis.

To^mut1^F—NLFN	5’ CCGGAGTCCAGCCACTACCATTTCAGTGCTCTGGCCGCTGGGGACAAGGCGC 3
To^mut1^R—NLFN	5’ GCGCCTTGTCCCCAGCGGCCAGAGCACTGAAATGGTAGTGGCTGGACTCCGG 3’
To^mut2^F—ADLF	5’ GGGGACAAGGCGCTGGGTGCCAACATGAATGTCGCCCTCAACGAAAACTCGGAGG 3’
To^mut2^R—ADLF	5’ CCTCCGAGTTTTCGTTGAGGGCGACATTCATGTTGGCACCCAGCGCCTTGTCCCC 3’

#### Amphipathic helix analysis

Sequences for all 23 members were aligned using the multi-align tool in NCBI and the region of the internal helix was selected based on the modelled Takeout structure. Each predicted helical region was then analyzed using HeliQuest (http://heliquest.ipmc.cnrs.fr/).

### Behavioral assays

Virgin males were collected within two hours of eclosion and housed separately in individual vials for 7 to 8 days at 25°C in a 12:12 light:dark cycle incubator. On the day of testing flies were acclimatized to room temperature (23°C) for two hours. The mating assays were performed in circular arenas with dimensions of 8mm (diameter) X 2mm. Virgin females were collected at least three hours before the assay. A single female was paired with a single male and all the steps of courtship (orientation, chase, wing extension, tapping, abdominal bending) were manually scored for 10 mins. 20 males per genotype were tested. The Courtship Index was calculated as the fraction of time a male performs any of the courtship steps within the 10-minute observation period.

### Statistical analysis

One-way ANOVA was performed with post-hoc Bonferroni test using Statview (Adept Scientifics, Bethesda, MD).

### Western blots

Transgene expression levels were assessed by Western blot. Protein was extracted from 5 male heads for each genotype, with three independent biological replicates. Samples were run on a 12% gel and transfer was carried out at 4°C, for 90mins at 90Volts onto a nitrocellulose membrane. The membrane was blocked with 4% Dry milk in TBST (50 mM Tris, 150 mM NaCl, 0.1% Tween 20) for an hour and washed thrice in TBST for 10 mins each. The membrane was incubated in 1% BSA in 1X TBST with 1:1000 diluted anti-V5 antibody (Invitrogen) overnight at 4°C. The membrane was washed three times for 10mins each and incubated with HRP-coupled goat anti-mouse secondary antibody for two hours, washed thrice for 10mins and imaged post incubation for 1min with HRP substrate solution mix in 1:1 ratio (Thermofisher-PierceTM).

## Results

### *takeout* homologues can rescue the courtship defect

The *takeout* gene family is conserved among all examined insects [13], suggesting that its members have important biological functions. Several have been implicated in biological functions, but besides *takeout*, little is known about the roles of different family members. Amino acid identities between *takeout* and its orthologs from its closest species i.e. *D*. *simulans* and *D*. *sechellia* are high (~above 95%), and as low as 18% amongst the most distant paralog (*CG16820*). This could indicate that the family members have diverged and assumed different functions. If this were the case, they would likely no longer be able to substitute for each other. Alternatively, they might be functionally conserved, but act in different tissues or at different times. To examine the functional conservation of *takeout* family members we decided to test the ability of several homologues to rescue the *D*. *melanogaster takeout* courtship defect. *D*. *mel*. *takeout* is male-preferentially expressed in the fat body and has a well described role in male courtship behavior [8]. We have shown that it acts in a genetic pathway with *fruitless (fru)*, a major courtship regulator. Mutant *takeout* males have reduced courtship, and when they are heterozygous for *fru* at the same time, courtship is further reduced with a courtship index around 0.5–0.6, whereas wildtype (wt) males have courtship indices of over 0.9. We used this sensitized mutant background to test the ability of *takeout* homologues to rescue the *takeout* courtship defect. We have shown earlier that expression of wildtype *takeout* in this genetic background rescues courtship [7]. In the experiments described below, we used our fat body driver *Lsp2-Gal4* [8] to express family members in the fat body of *to*^*1*^*/to*^*1*^;*fru*^*4*^*/+* mutants.

**Fig 1:**
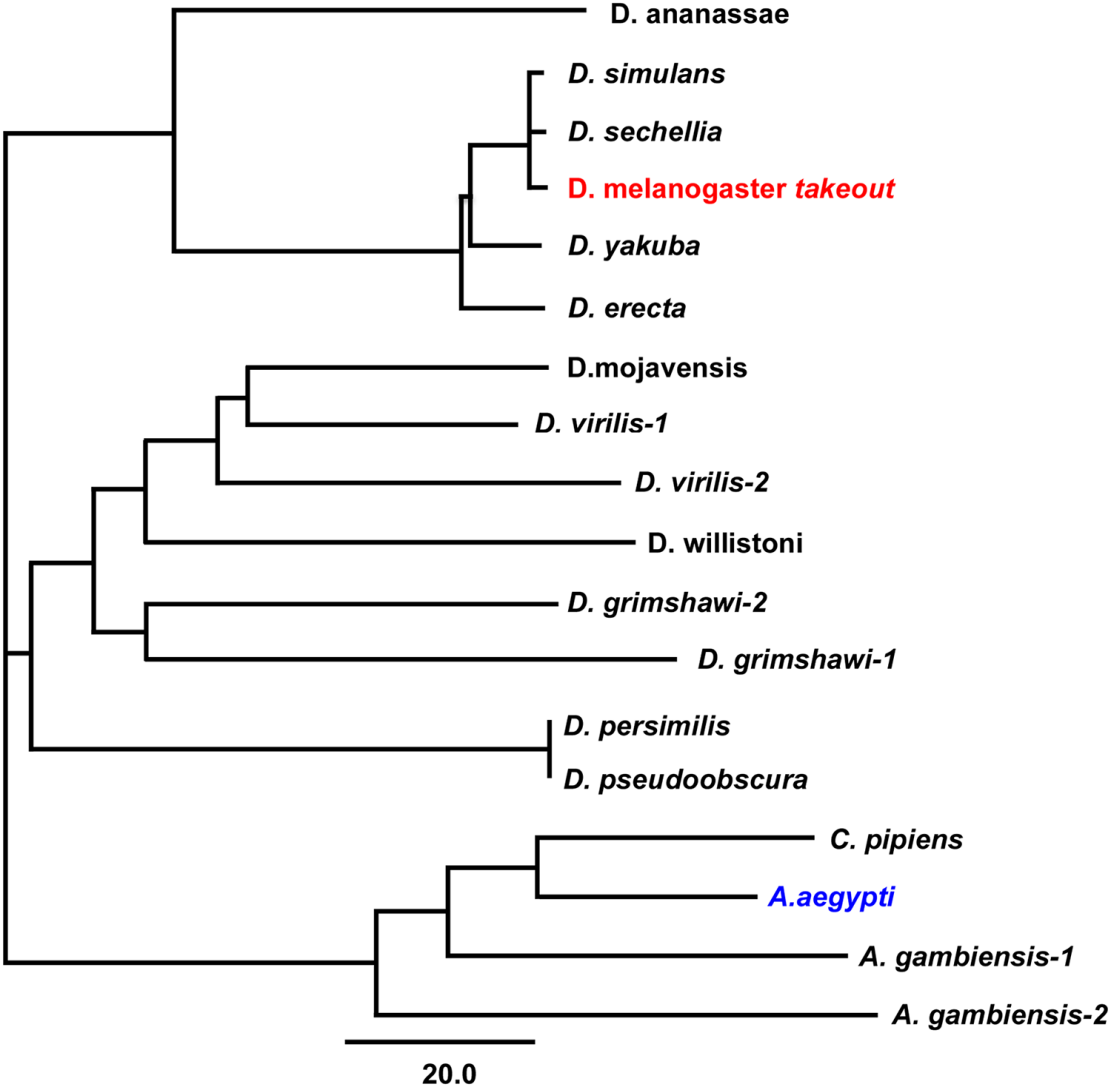
Phylogenetic tree representation of the Takeout cluster. This cluster contains *D*.*melanogaster* Takeout (*CG11853*) and the cluster members from the indicated insect species. The *A*. *aegypti* member that was tested for functional complementation analysis is indicated in blue. The scale bar represents 20 nucleotide substitutions per site.

Our earlier studies [13] have identified the *Aedes aegypti takeout* homologue (AAEL011966) within the *Aedes takeout* family (Fig 1). It is 42.6% identical and 59.9% similar to *D*. *melanogaster takeout*.

**Fig 2:**
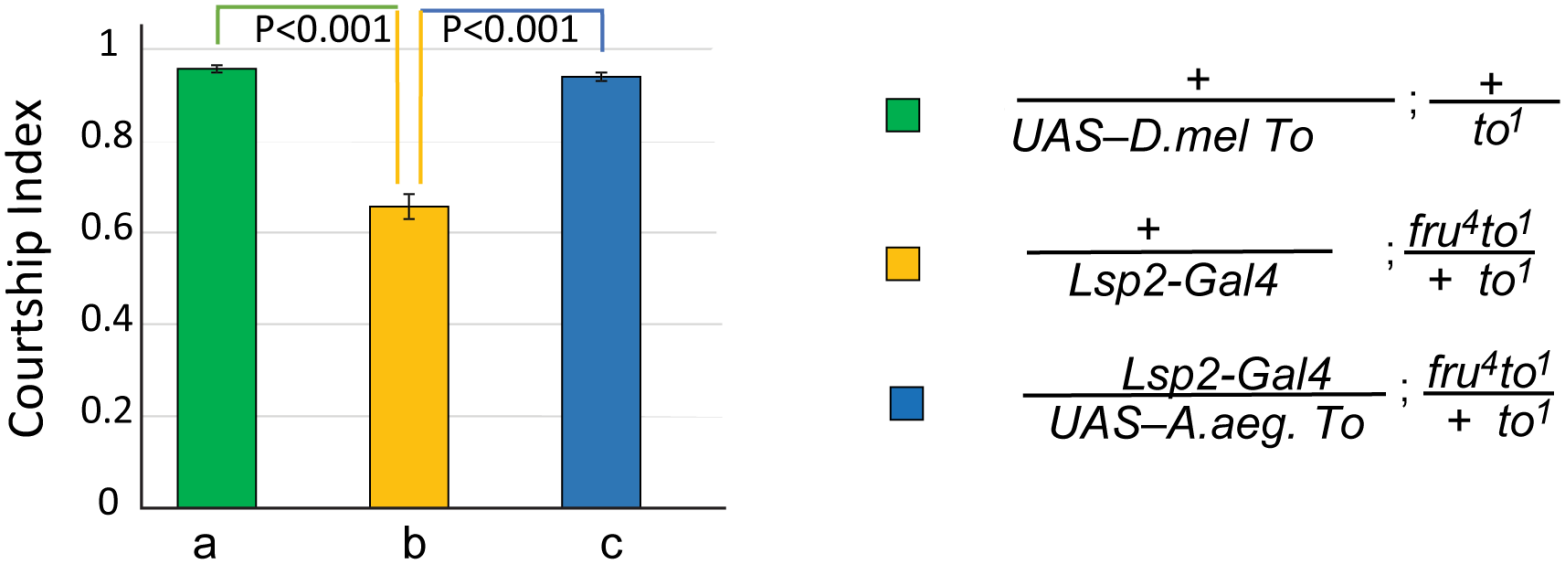
*A*. *aegypti* Takeout can functionally rescue the courtship defects *of D*. *melanogaster to*^*1*^ mutants. Graph shows the courtship index CI (fraction of time males spend courting during the observation period) ± SEM of the indicated genotypes. The *Lsp-2-Gal4* fat body driver was used to express the *A*. *aegypti* To transgene in a *fru*^*4*^*to*^*1*^*/to*^*1*^ mutant background (b, yellow, CI 0.66±0.03). *A*. *aegypti* To rescues the behavior (p<0.001). There was no significant difference between positive control (a, green, CI 0.96±0.01) and the rescue males (c, blue, CI 0.94±0.01). Data were analyzed by ANOVA followed by *Bonferroni multiple comparisons (N = 20)*.

To test whether *Aedes takeout* is capable of rescuing the *Drosophila takeout* courtship defect, we created an *UAS-Aedes-takeout* transgene by amplifying the sequence from a *A*. *aegypti* male RNA library kindly provided by Dr. David Severson, University of Notre Dame. The RNA had been isolated from the strain that was used for the *Aedes aegypti* genome sequences [23] in which we identified the homologue. We observed complete rescue when the *Aedes takeout* homologue was expressed in the fat body of *to*^*1*^*/to*^*1*^;*fru*^*4*^*/+* mutants (Fig 2). This experiment shows that *A*. *aegypti takeout* can functionally complement for *D*. *melanogaster takeout* when expressed in the fat body. Thus, despite low sequence conservation, these two proteins are capable of interacting with the same courtship pathways and might bind the same putative ligand.

**Fig 3:**
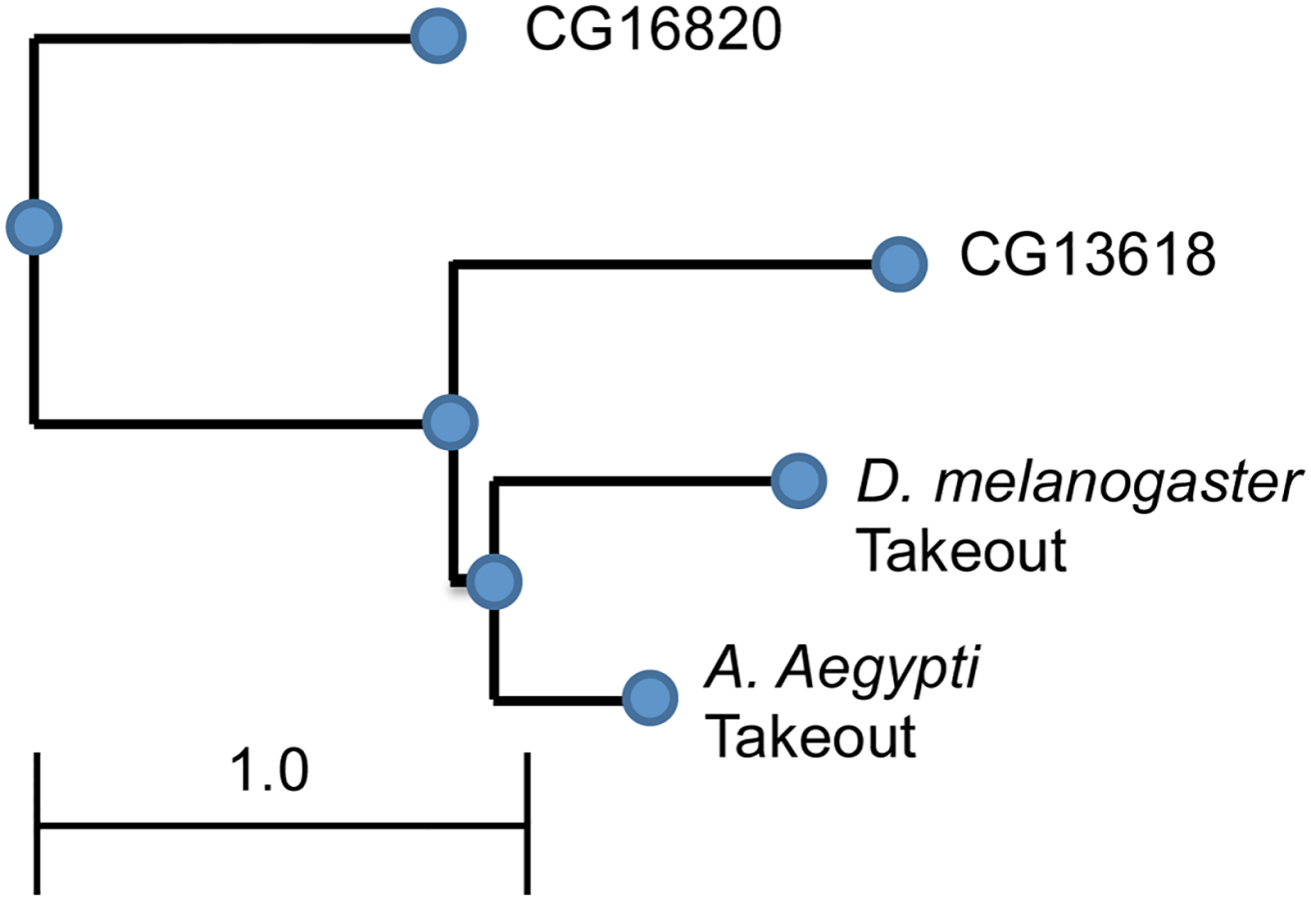
Phylogenetic tree analysis of candidate genes chosen for functional complementation analysis. *D*. *mel*. Takeout, *D*. *mel takeout* family members *CG13618* and *CG16820*, and *A*. *aegypti* To are compared. Scale bar length represents number of amino acid substitutions per site.

These findings raise the question whether this exchangeability is limited to *takeout* homologues, or whether other members of the *D*. *melanogaster takeout* family are similarly able to substitute for *takeout*. We chose two *D*.*mel*. family members that belong to two separate clusters, *CG13618* and *CG16820* [13]. Their relationship to *D*. *mel takeout* and *A*.*aegypti takeout* is shown in Fig 3. Both *CG13618* and *CG16820* are significantly less similar to *D*.*mel takeout* than *A*.*aegypti takeout* is.

**Fig 4:**
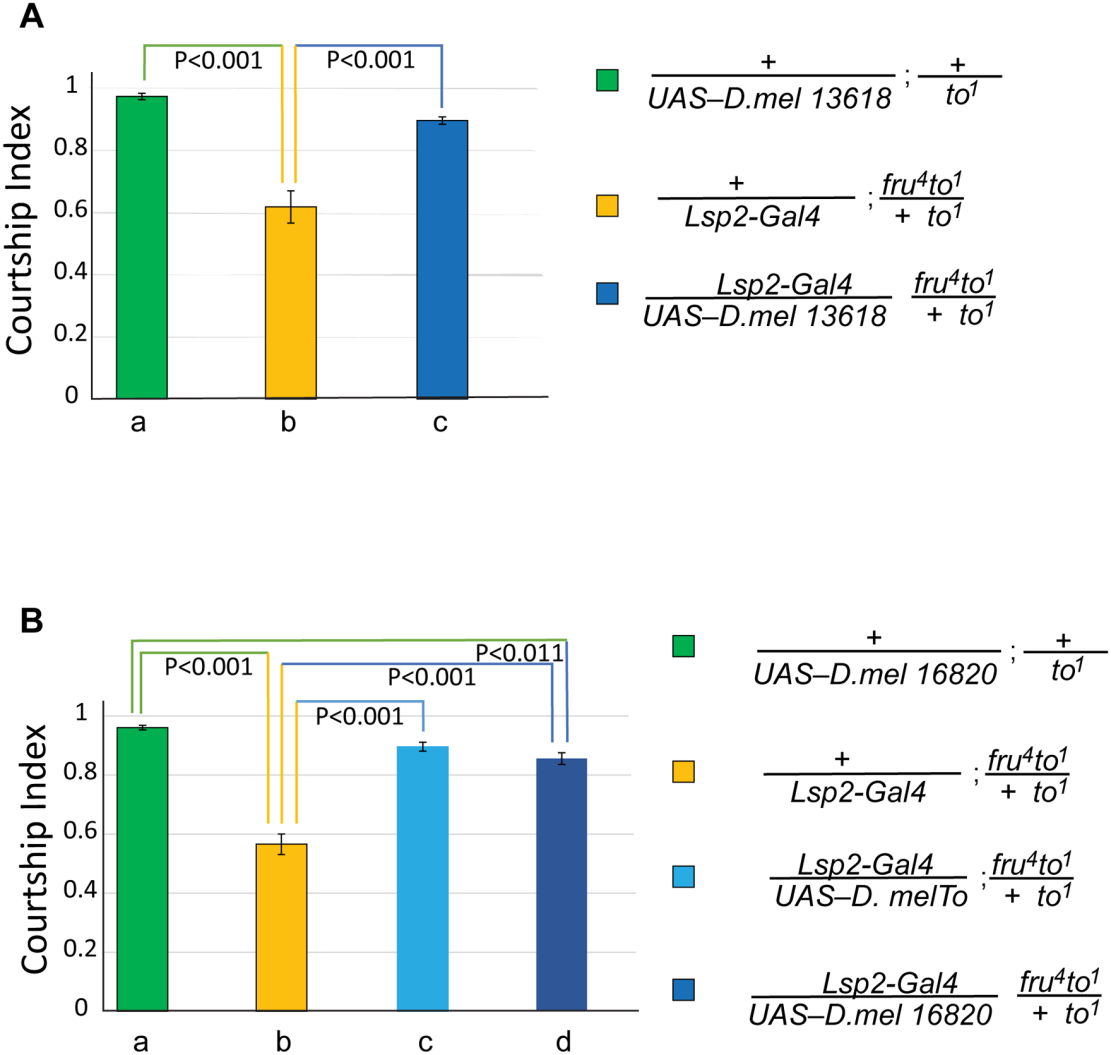
*D*. *melanogaster* paralogs *CG13618* and *CG16820* can rescue the courtship defects of *D*. *melanogaster to*^*1*^ mutants. Graph shows the courtship index CI (fraction of time males spend courting during the observation period) ± SEM of the indicated genotypes. (A) *CG13618* is a *D*. *mel* To paralog outside the To cluster and has 51.4% similarity to To. Male flies heterozygous for *to*^*1*^ show a robust courtship (a, green, CI 0.98±0.01). *to*^*1*^ homozygous flies with a *fru*^*4*^ heterozygous genetic background show severely reduced courtship (b, yellow, CI 0.62±0.05). Expressing *CG13618* in the mutant strain using the *Lsp-2-Gal4* fat body driver completely rescues the mutant phenotype (c, blue, CI 0.90±0.01). (B) *CG16820* is a *D*. *mel* To paralog outside the To cluster with 35.3% similarity to To. It rescues the mutant phenotype (d, blue, CI 0.86±0.02), similar to a *D*. *mel to* wildtype transgene (c, light blue, CI 0.90±0.02). *to*^*1*^ homozygous flies with a *fru*^*4*^ heterozygous genetic background show severely reduced courtship (b, yellow, CI 0.57±0.04). Data were analyzed by ANOVA followed by *Bonferroni multiple comparisons (N = 20)*.

Again, we expressed the transgenes in the fat body of *to*^*1*^*/to*^*1*^;*fru*^*4*^*/+* mutant males and tested their courtship behavior (Fig 4a and 4b). We observed complete rescue with *UAS-CG13618*. For *UAS-16820* there was no significant difference between the rescue shown by flies expressing wildtype *takeout* and *UAS-CG16820*. In comparison with *CantonS* wild type flies, however, flies expressing *CG16820* scored lower than the flies with the wildtype transgene. This suggests that although the rescue was significant it was not as robust as the rescue shown by flies expressing *takeout*.

### Takeout members share structural features

**Fig 5:**
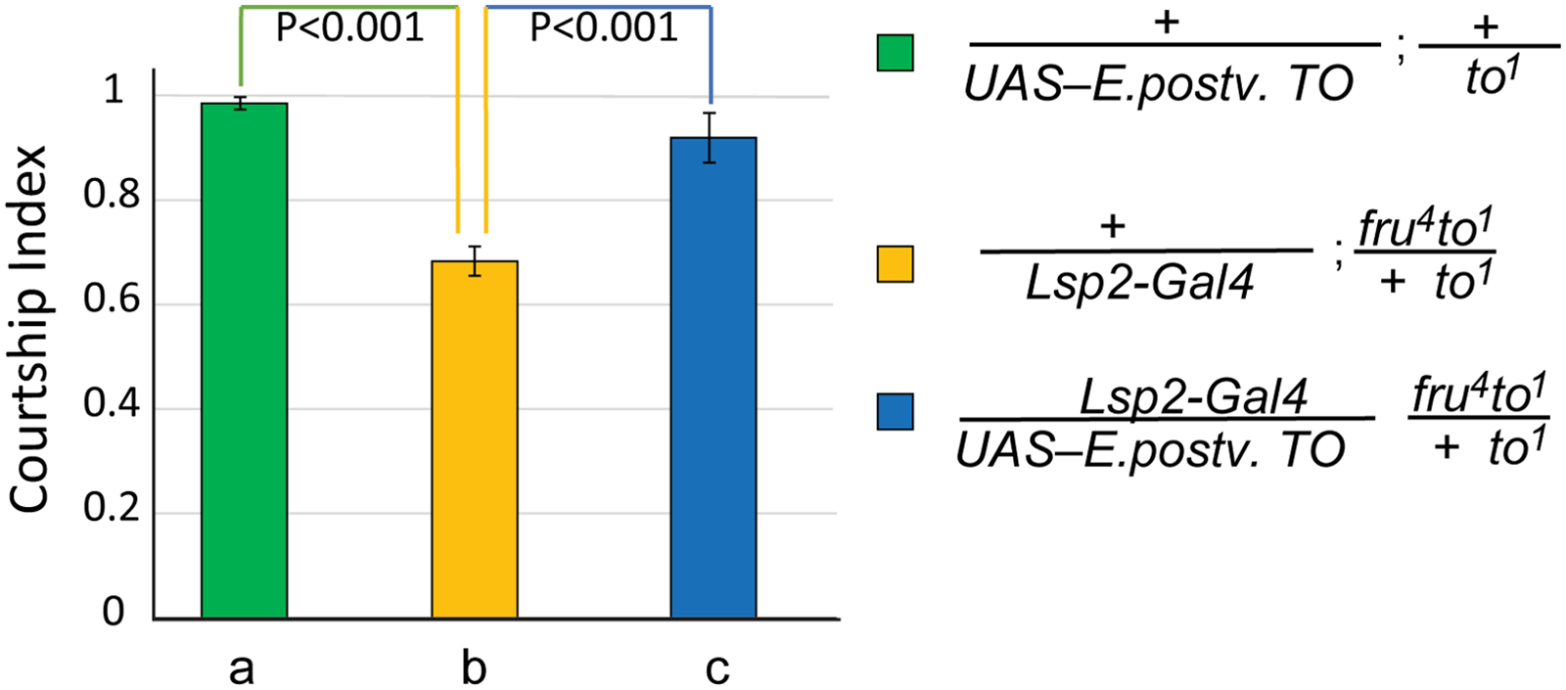
*E*. *postvittana* Takeout can functionally rescue the courtship defects *of D*. *melanogaster to*^*1*^ mutants. Graph shows the courtship index CI (fraction of time males spend courting during the observation period) ± SEM of the indicated genotypes. The *Lsp-2-Gal4* fat body driver was used to express the *E*.*postvittana TO* transgene in a *fru*^*4*^*to*^*1*^*/to*^*1*^ mutant background. *E*.*postvittana* To rescues the behavior (p<0.001). There was no significance difference between positive control (a, green, CI 0.92±0.05) and the rescue males (c, blue, CI 94±0.01). *Lsp-2-Gal4/+; fru*^*4*^, *to*^*1*^*/to*^*1*^ CI = 0.68±0.03. Data were analyzed by ANOVA followed by *Bonferroni multiple comparisons (N = 20)*.

These data show that several *takeout* family members can substitute for *D*.*mel takeout* in courtship. Rescue is observed despite low sequence similarity, with members from within *D*. *melanogaster* and from across species. This suggests that these proteins share structural features that are critical for their function. The protein structure for *D*.*mel* Takeout has not been determined, but the structure of a *takeout* family member from *Epiphyas postvittana* (Light Brown Apple Moth, an agricultural pest) has been solved [19, 21]. Interestingly, this particular *E*. *postvittana takeout* relative is expressed at higher levels in male antennae than in female antennae [7]. The *E*. *postivittana takeout* relative is most similar to *D*. *melanogaster* homologs *CG2650* (96% query coverage, 27% identical), and second most similar to *CG10264* (95% query coverage, 27% identical). *Epiphyas* Takeout has been crystallized as both a bacterially expressed protein, and following expression in insect cells in a baculovirus system [19, 21]. Interestingly, in both cases the protein co-crystallized with a ligand bound in a large internal cavity (ubiquinion in the bacterial system, and fatty acids in the insect cells). Under both conditions the proteins acquired a nearly identical crystal structure, suggesting robust structural features of the protein [19, 21]. To obtain an understanding of potentially unifying structural features across species, we decided to model *D*. *melanogaster* Takeout onto the *Epiphyas* structure. But first, we examined whether *E*. *postvittana takeout* was capable of rescuing the *takeout* courtship phenotype. We found that the *Epiphyas* sequence fully rescued, indicating that it possesses the critical Takeout characteristics required for courtship (Fig 5).

We used the *E*. *postvittana structure* as the template to model *D*. *melanogaster Takeout*. The sequence identity between the *E*. *postvittana takeout* homolog and *takeout* is 31%. This is close to the minimum required sequence identity for a good 3D model. The sequence coverage was about 90%, sufficient for use as a template. We used the Swiss Homology modeling online server (http://swissmodel.expasy.org/) for Homology Modeling. *E*. *postvittana* 3E8T was used as the template. We retrieved the structure with best QMEAN score values (QMEAN–Quality Model Energy Analysis Score) [24]. The modeled PDB structure was downloaded and visualized with the help of the protein visualization software CHIMERA. Most residues were found in favorable regions as assessed by Ramachandran Plot analysis by the MOLPROBITY server http://molprobity.biochem.duke.edu [22].

**Fig 6:**
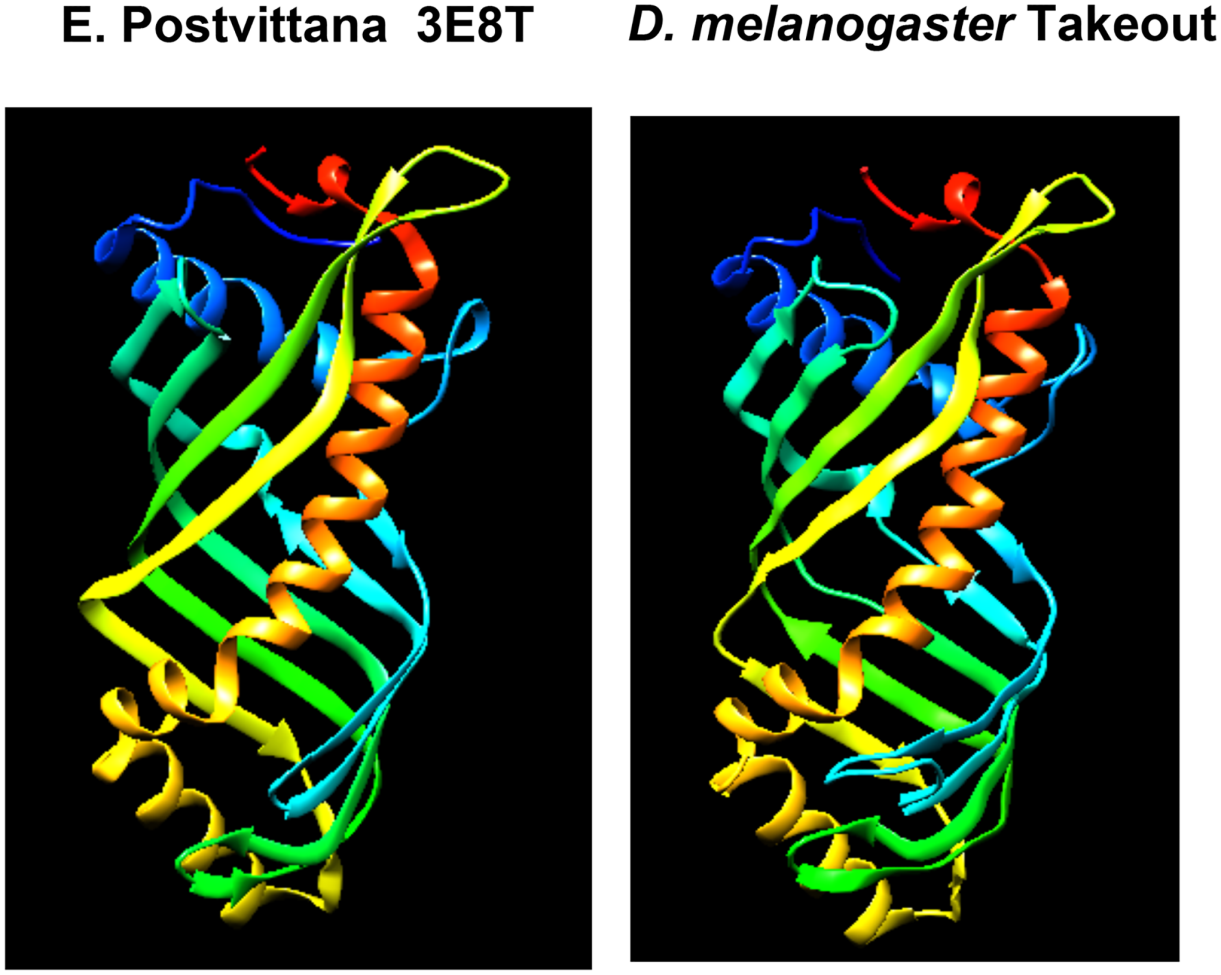
Homology modeling of *D*. *melanogator* TO onto the *E*. *postvittana* Takeout crystal structure (3E8T). The *D*. *mel* To sequence was modeled onto the structural template of the *E*. *postvittana* crystal structure (PDB ID– 3E8T) using the SWIZZ homology modeling server http://swissmodel.expasy.org/. The quality of the structure and residue conflicts were evaluated using MOLPROBITY (Ramachandran Plot Analysis). Structures are rendered in CHIMERA. 95.5% of all residues were found in favorable regions.

As shown in Fig 6, *D*. *mel* Takeout could be modeled well onto the *E*. *postvittana* structure. Despite only 31% BLAST identity, there is broad agreement in the structure of the two proteins. EpTo1 adopts the so-called TULIP fold [25] that consists of a long alpha-helix wrapped into a curved anti-parallel beta-sheet. The space between the helix and the sheet forms a long internal cavity that allows the binding of the co-crystallized ligands [19, 21]. As seen in Fig 6, the same structural features are observed in the modeled *D*. *melanogaster* Takeout structure.

**Fig 7:**
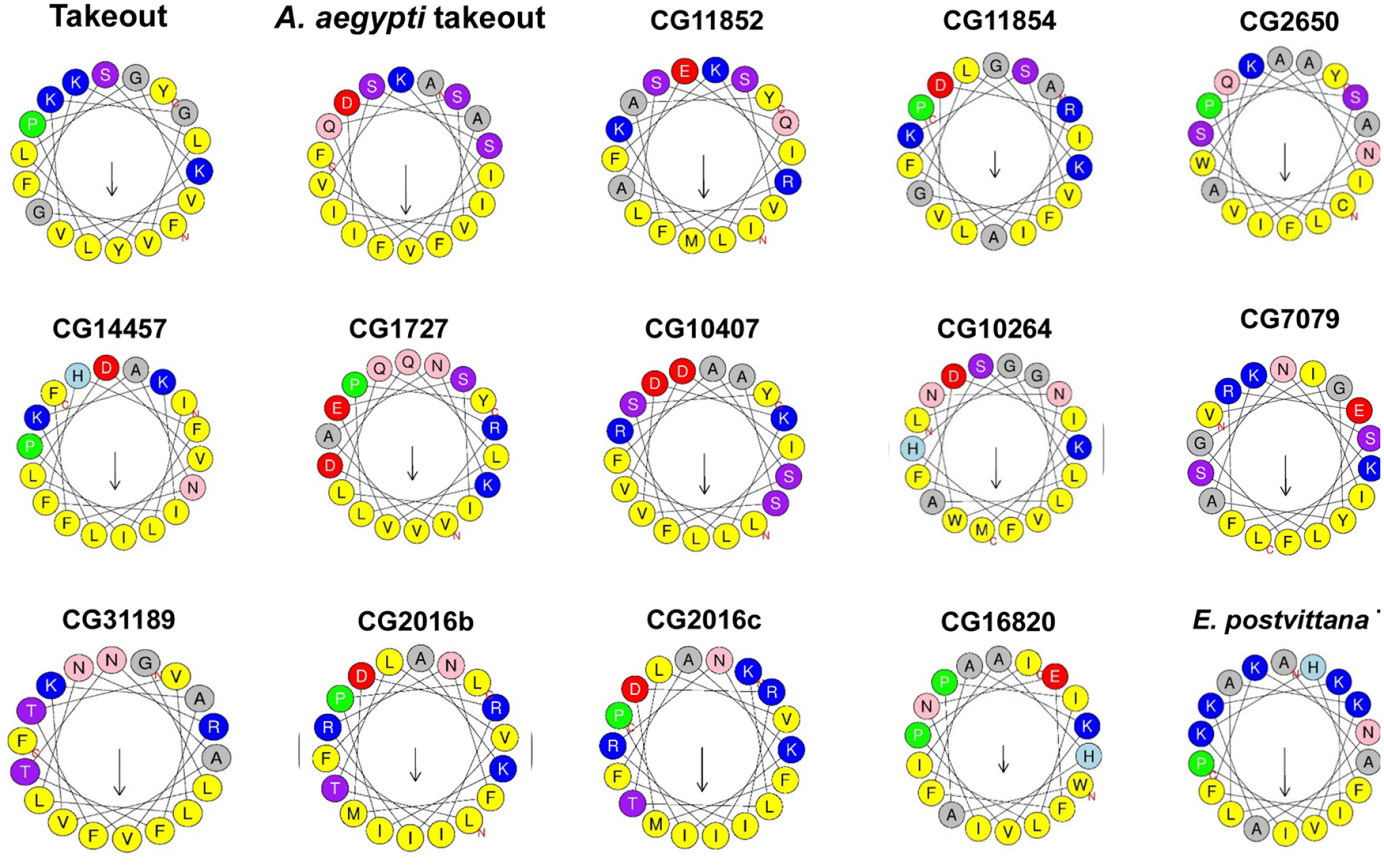
Takeout family members share an amphipathic C-terminal helix. *D*. *mel* To and family members were aligned using PRALINE, and the sequence of the C-terminal helix extracted and analyzed by HELIQUEST. All members show hydrophobic residues aligned towards the cavity.

The cavity of *E*. *postvittana* Takeout is lined with side chains of hydrophobic residues that are located in the β-sheets and the α-helix that surround the cavity. This cavity is likely important in the binding of putative ligands (as indeed both E. postvittana proteins co-crystallized with (different) ligands), and we were curious whether this characteristic might be shared by *takeout* family members. Based on the *E*. *postvittana* crystal structure, the sequence of the alpha helix was retrieved from different To homologues and analyzed for amphipathic nature using the online server HELIQUEST (http://heliquest.ipmc.cnrs.fr/) [26]. As shown in Fig 7, most To homologues have an amphipathic helix. Hydrophobic residues are shown in yellow. Remarkably, they line up on one side of the helix facing the cavity, suggesting that the cavity could accommodate ligands(s) with both hydrophobic and hydrophilic domains. Together these findings support our hypothesis that the *takeout* family members share a number of structural features that are highly conserved and that likely account for their conserved functional properties in courtship.

### Amino acids in motifs 2 are required for Takeout function

**Fig 8:**
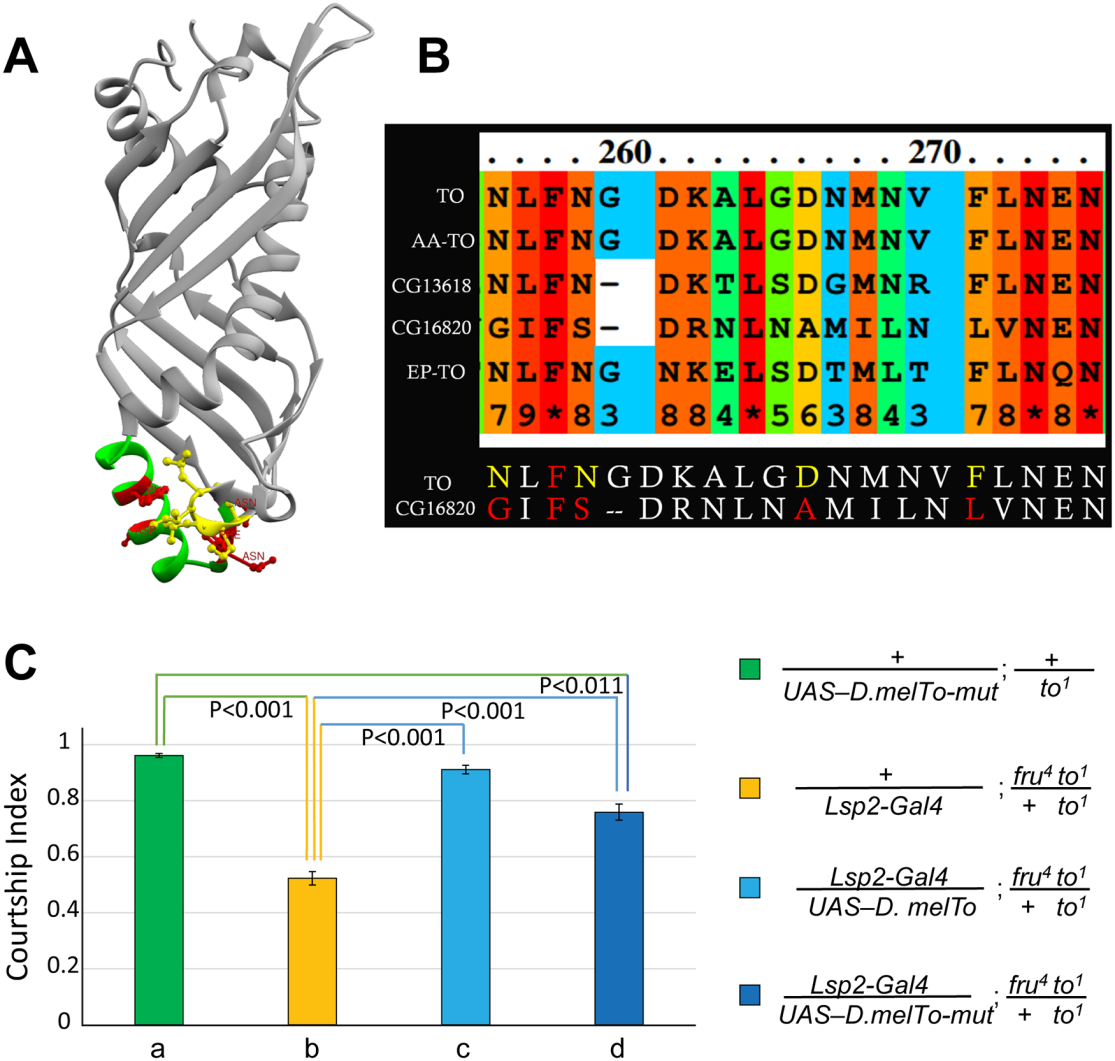
Amino acids in motifs 2 are required for Takeout function. (A) Conserved motifs 1 (yellow) and 2 (red) are exposed at the bottom of the protein in the *D*. *mel* model (A), separated by helix 2 (green). Residues that are at the bottom of the protein and predicted to be exposed were considered for site directed mutagenesis in *D*. *mel* TO. (B) Alignment of Motif 2 in all tested To homologs using PRALINE is shown. Conservation scores generated by the program are color coded, and a conservation index is indicated underneath each residue. Note that *CG16820* shows several deviations from the consensus in this region. Residues colored in yellow were mutated to Alanine, including a Phenylalanine at position 258 that was conserved in all sequences, creating *TO-mut*. (C) *TO-mut* only partially rescues the *takeout* courtship defect. Graph shows the courtship index CI (fraction of time males spend courting during the observation period) ± SEM of the indicated genotypes. The *Lsp-2-Gal4* fat body driver was used to express *TO-mut* in a *fru*^*4*^*to*^*1*^*/to*^*1*^ mutant background. Male flies heterozygous for *to*^*1*^ show robust courtship (a, green, CI 0.96±0.01). *to*^*1*^ homozygous flies with a *fru*^*4*^ heterozygous genetic background show severely reduced courtship (b, yellow, 0.52±0.02). While a *To* wildtype transgene rescues the behavior (c, light blue, CI 0.91±0.02), expression of *TO-mut* only shows partial rescue (d, blue, 0.76±0.03). Data were analyzed by ANOVA followed by Bonferroni multiple comparisons *(N = 20)*.

As described previously [12], two motifs (motif 1 and motif 2) in the *takeout* family of proteins are more conserved than the rest of the proteins in the gene family. Fig 8A shows the location of these domains in the modeled *D*. *mel*. To structure. In the EpTo1 structure, motif 2 is exposed at the bottom end of the barrel. Hemiaux et al. [19] suggest that motifs 1 and 2, together with the helix, contribute to the observed stable structure of the protein. Since residues in motif 2 were hydrophilic and localized at the bottom we hypothesized that these might enable interactions of Takeout with other proteins. Fig 8 shows this region in the Takeout homologs we tested. Note that 16820, the protein with the least robust rescue, shows several deviations from the consensus in this region. To test the hypothesis that residues in this region are functionally significant for Takeout function, we exchanged the amino acids indicated in yellow and red with Alanine residues. We then tested the mutant protein in our courtship rescue assay. We found that it was unable to completely rescue the courtship defect, indicating a role for these residues in the regulation of courtship (Fig 8C).

### The Moth Juvenile Hormone Binding protein can only partially substitute for Takeout

**Fig 9:**
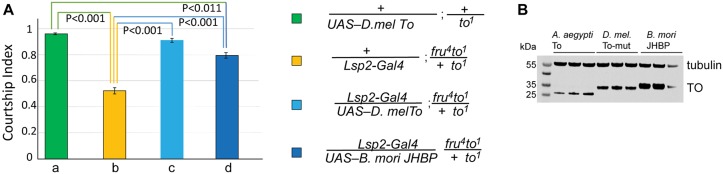
*B*. *mori* Juvenile Hormone Binding protein (JHBP) partially rescues the courtship defects of *D*. *melanogaster to*^*1*^ mutants. (A) Graph shows the courtship index CI (fraction of time males spend courting during the observation period) ± SEM of the indicated genotypes. The *Lsp-2-Gal4* fat body driver was used to express *B*. *mori* JHBP in a *fru*^*4*^*to*^*1*^*/to*^*1*^ mutant background. Male flies heterozygous for *to*^*1*^ show a robust courtship (a, green, CI 0.96±0.01). *to*^*1*^ homozygous flies with a *fru*^*4*^ heterozygous genetic background show severely reduced courtship (b, yellow, CI 0.52±0.02). While a To wildtype transgene rescues the behavior (c, light blue, CI 0.91±0.02), expression of *B*. *mori* JHBP only shows partial rescue (d, blue, CI 0.80±0.02). Data were analyzed by ANOVA followed by Bonferroni multiple comparisons *(N = 20)*. (B) Western blot showing three independent biological samples each of the genotypes indicated. V5-tagged Takeout proteins were visualized with anti-V5 antibody. Alpha-Tubulin is shown as loading control.

Given the observed ability of Takeout family members to substitute for each other, we were curious whether this would extend to a related family of proteins. Takeout is most similar to Juvenile Hormone Binding Proteins (JHBPs) [9, 27]. While JHBPs have been identified and characterized in many insect species, they have not been found in *D*. *melanogaster*. We wondered whether a well-characterized JHBP from *Bombyx mori* would be able to rescue the *takeout* courtship defect. We obtained *B*. *mori* JHBP cDNA from Dr. Toshimasa Yamazaki and made a *UAS-B*.*mori -JHBP* transgene. As shown in Fig 9, *Bombyx* JHBP only partially rescued the *takeout* courtship deficit. This difference in rescue is not due to lower levels of protein expression (Fig 9B). These results indicate that Takeout and *B*.*mori* JHBP are not interchangeable in *D*. *melanogaster* courtship, but that they do share some conserved features that allows partial rescue.

## Discussion

We show here that distant To homologs and other members of the *takeout* gene family can functionally substitute for *D*.*mel* To in male courtship behavior despite their low sequence identity. We observed functional rescue with To from two different species, *A*. *aegypti* (Dipteran) and *Epiphyas postvittana* (Lepidopteran), and with two To paralogs, CG16820 and CG13618. Our phylogenetic analysis had placed *D*. *mel* To and the tested *A*. *aegypti* To in the same orthologous cluster [13], prompting us to ask whether they might be functionally conserved. We found that *A*. *aegypti* To was indeed able to fully complement for *D*. *mel*. To in *Drosophila* male courtship behavior. Their overall sequence similarity is greater than that found among *D*. *melanogaster* family members themselves. This raised the question whether members of a specific cluster might possess functional properties not shared by other clusters. The results presented here suggest broad functional conservation among family members from different species and clusters when tested for courtship.

**Fig 10:**
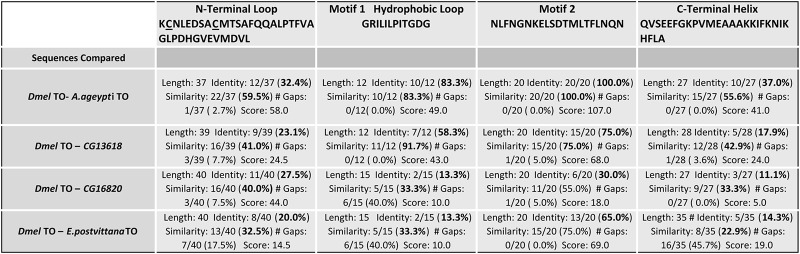
Takeout protein domains vary in their conservation. Protein sequences of *D*. *mel*. TO and tested TO family members were compared in a pairwise fashion using EMBOSS Needle (ebi.ac.uk). Domains were chosen based on sequence comparison and potential functional significance, in reference to the known *E*. *postvittana* structure. The N-terminal loop is the region adjacent to the first helix. The sequence contains two cysteine residues (underlined) that are present in all family members. Motifs 1 and 2 are the two most conserved domains of the proteins and likely important structural features. The C-terminal helix could potentially interact with the N-terminus to affect ligand capturing.

As shown in Fig 10, while overall conservation of the tested proteins is low, specific domains show higher conservation. Conservation is highest in motif 2. It is 100% for *A*. *aegypti* Takeout, consistent with the fact that *D*. *mel* TO and the *A*. *aegypti* TO we chose for this experiment belong to the same cluster within the family. Since motif 2 is the most conserved domain among the proteins it is likely that its conservation is a major reason why all family members we tested were capable of rescuing the *takeout* courtship phenotype. In agreement with this, the “marginally complete” rescue ability of *CG16820* correlates with its lower similarity in motif 2. The importance of motif 2 is underscored by our experimental finding that mutations of conserved residues in this motif compromised protein function. The proteins of the entire To family are likely secreted proteins since they all have a putative signal sequence. Indeed, we expressed the proteins we tested in the fat body, from where they were likely secreted into the hemolymph to effect their rescue.

Our findings speak to the similarities among the *takeout* family proteins, and identify an important domain of the protein, but they do not answer the question why there are so many family members, and why the family is conserved across insect species. Their structure and the fact that *E*. *postvittana* co-crystallized with two different ligands suggests that they bind ligands. These ligands may vary in a tissue-specific manner and reflect the local cellular environment, and determine the degree to which Takeout proteins can exert their function. However, if the family members carry different ligands, they’re likely to not do so in an exclusive manner, since they were all active in the fat body/hemolymph environment to support courtship. As shown here, the *D*. *mel*. Takeout structure can be very closely modeled onto the structure of a known *Epiphyas* Takeout family member, further supporting the previously described robustness of structure most likely conferred by the two alpha helices and a beta sheet. Crystallization studies and the nature of the residues lining the cavity indicate that these proteins can bind hydrophilic ligands with both hydrophilic and hydrophocic characteristics. Takeout expressed in a baculovirus system co-crystallized with a mixture of fatty acid moieties, mostly myristic and palmitic acid bound inside the EpTo1 cavity. The natural ligand(s) of the TO proteins will therefore likely have structural similarity to the ligands that were found in the experimental systems. Similar fatty acids might be the natural ligands for To proteins. Although *takeout* family members are most similar to Juvenile Hormone Binding Proteins (JHBPs), it is not known whether they are capable of binding JH. If they were, it would be tempting to speculate that the large number of TO members could reflect diverse functions as specific JH binding proteins. While similar in structure, important differences exist between the two kinds of proteins [19]. As our experiments show, *B*. *mori* JHBP can partially substitute for *takeout* in courtship, but can not rescue fully, underscoring the difference between the two proteins. In many insect species, both JHBP and Takeout family members are present, but not in *Drosophila* where JHBPs have not been found and the only established JH binding proteins are intracellular receptors with characteristics of transcription factors [28–30]. It is unknown whether To can adopt the role of JHBP in *D*. *melanogaster* and serve to protect JH from degradation and target the hormone to specific cells.

Another possibility is that family members act locally and their specific site of expression contributes to specific functions. Where individual family members are expressed and function is largely unknown, although a number of them were identified when antennal transcripts were analyzed. The functional significance of these findings is unknown. Since *to* mutants can be fully rescued by expression of the wildtype protein in the fat body, antennal *takeout* expression does not appear to be required for courtship. The functional significance of *takeout* expression in the antennae has not been established yet. To family members from other species have been documented in antennae and labellum [18, 31–34], often in a male-enriched fashion. For example, the *A*. *aegypti* To orthologue was found to be enriched in male antennae [31]. Takeout family members have been implicated in a number of physiological processes, such as feeding behavior [9], gustatory perception in response to starvation [20], as well as increased feeding activity and olfactory sensitivity in female mosquitoes [34]. To RNA and protein levels were found to be under circadian control in at least two different species of Diptera, *D*. *melanogaster* [9, 12, 16] and *Anopheles gambiae* [34].

Our results suggest that the Takeout family of proteins, despite overall low sequence identity, share functional properties that are largely determined by highly conserved structural features and functional conserved domains. Future studies characterizing the function of individual family members and identifying their natural ligand(s) will be required to understand the role of this family of proteins.
